# Supplementary material for: Phytotoxin synthesis genes and type III effector genes of Pseudomonas syringae pv. actinidiae biovar 6 are regulated by culture conditions
Source: PeerJ. 2020 Aug 14;8:e9697. doi: 10.7717/peerj.9697 (PMC7430302; doi:10.7717/peerj.9697)
Supplement: Supplemental Information 1 — Amplicons using PCR primers designed in this study were confirmed to be single bands by electrophoresis. A, The photographs of Psa6 amplicons. B, The photograph of Psa1 amplicons. C, The photograph of Psa3 amplicons. D, The photograph of Psg amplicons. [file peerj-08-9697-s001.pdf]

Supplemental Figure S1. The results of electrophoresis of amplicons.

Amplicons using PCR primers designed in this study (Table S1) were confirmed to be single bands by electrophoresis. **A,** The photographs of Psa6 amplicons. **B,** The photograph of Psa1 amplicons. **C,** The photograph of Psa3 amplicons. **D,** The photograph of Psg amplicons.

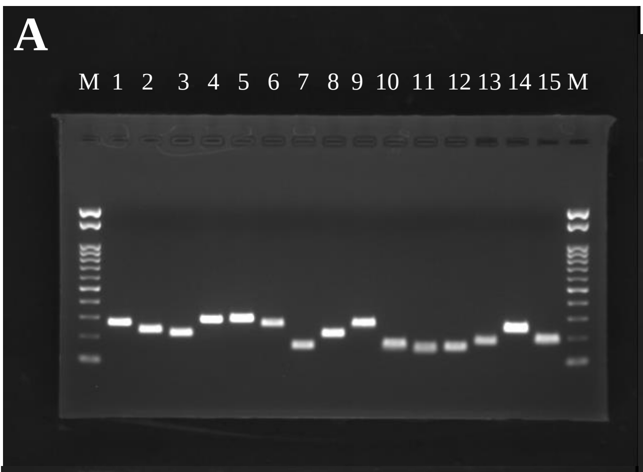

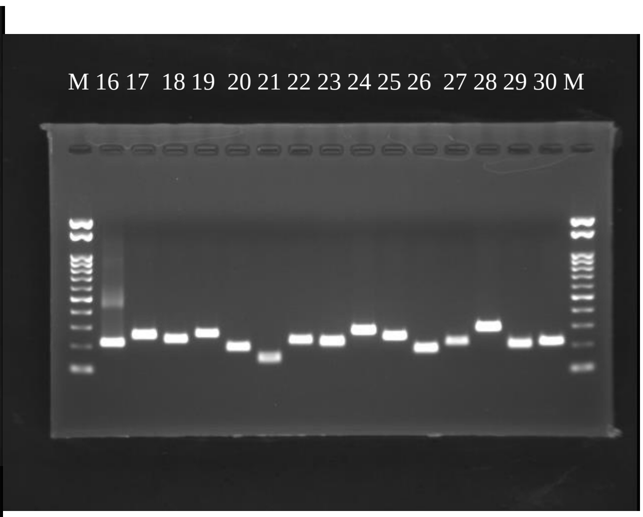

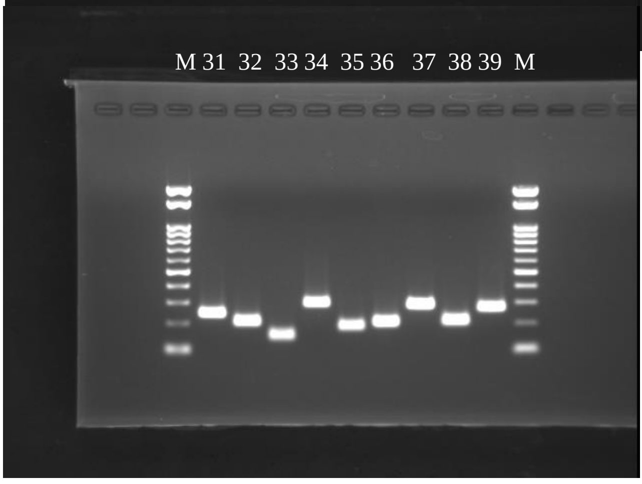

Lane
M: 100bp ladder marker
1: 50S ribosomal protein L13
2: bifunctional glutamine synthetase adenylyltransferase/deadenyltransferase
3: transcriptional regulator *ftrA*
4: microcin ABC transporter ATP-binding protein
5: tRNA dihydrouridine(20/20a) synthase *dusA*
6: *cfl*
7: *cfa1*
8: *cfa5*
9: *cfa9*
10: *cmaU*
11: *cmaD*
12: *corR*
13: *corS*
14: *corP*
15: *argK*
16: *argD*
17: *avrRps4*
18: *hopJ*
19: *hrpK*
20: *avrRpm1*
21: *hrpL*
22: *avrE1*
23: *hopM1*
24: *hopAA1-1*
25: *hopN1*
26: *hopS2*
27: *avrD1*
28: *hopD1*
29: *hopQ1*
30: *hopY1*
31: *hopZ3*
32: *hopAS1*
33: *hopAE1*
34: *hopR1*
35: *hopAH1*
36: *hopAI1*
37: *avrpto5*
38: *hopAZ1*
39: *hopAU1*

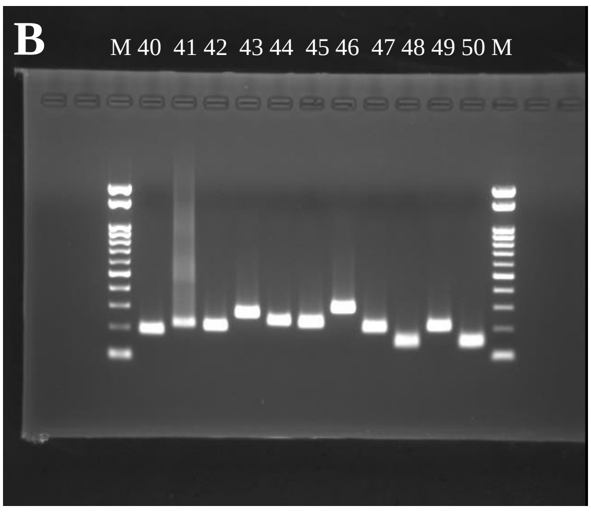

Lane
M: 100bp ladder marker
40: *argK*
41: *argD*
42: *hopAS1*
43: *hrpK*
44: *avrE*
45: *hopM1*
46: *hopD1*
47: *hopQ1*
48: *hopAE1*
49:*hopAZ1*
50: *hrpL*

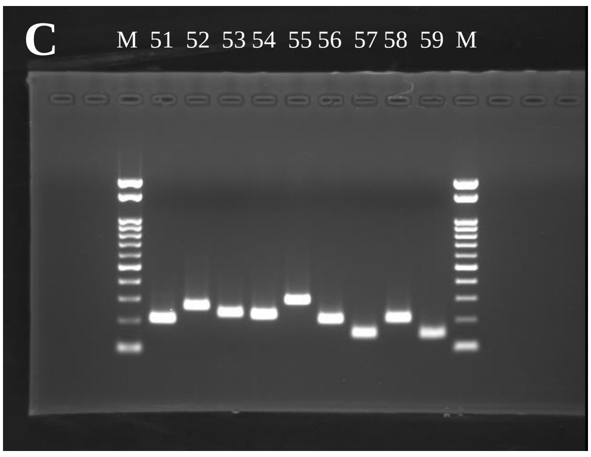

Lane
M: 100bp ladder marker
51: *hopAS1*
52: *hrpK*
53: *avrE*
54: *hopM1*
55: *hopD1*
56: *hopQ1*
57: *hopAE1*
58:*hopAZ1*
59: *hrpL*

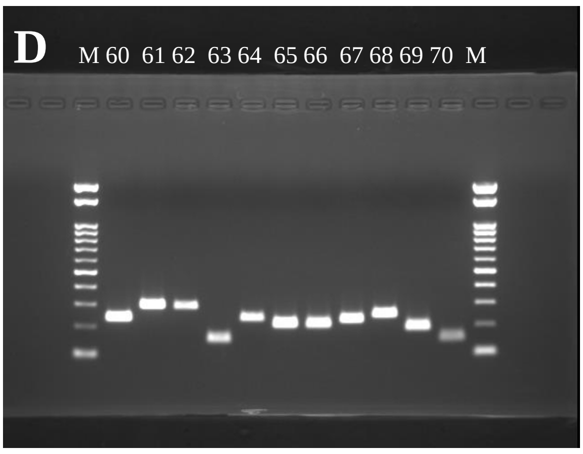

Lane
M: 100bp ladder marker
60: bifunctional glutamine synthetase adenylyltransferase/deadenyltransferase
61: tRNA dihydrouridine(20/20a) synthase *dusA*
62: microcin ABC transporter ATP-binding protein
63: *hrpL*
64: *avrE1*
65: *hopAS1*
66: *hopQ1*
67: *hopM1*
68: *hrpK*
69: *hopAZ1*
70: *hopAE1*
