# Supplementary material for: Phytotoxin synthesis genes and type III effector genes of Pseudomonas syringae pv. actinidiae biovar 6 are regulated by culture conditions
Source: PeerJ. 2020 Aug 14;8:e9697. doi: 10.7717/peerj.9697 (PMC7430302; doi:10.7717/peerj.9697)
Supplement: Supplemental Information 2 [file peerj-08-9697-s002.pdf]

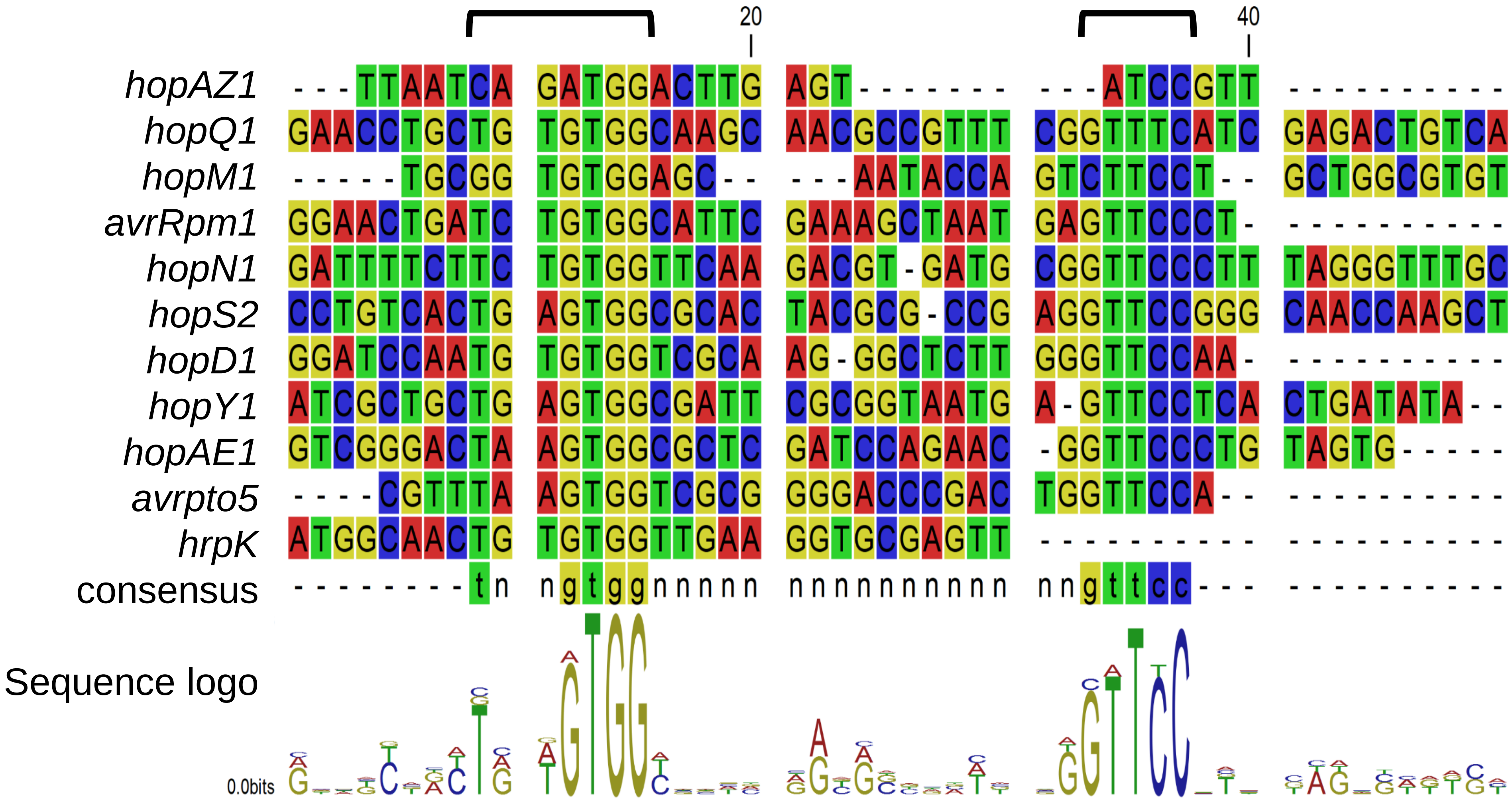

Supplemental Figure S2. The candidate Hrp box promoter sequences.
Th candidate Hrp box promoter sequences (consensus) were found in the upstream of the early inducible genes of Psa6.
